# Underlying Factors Explaining Physical Behaviors among Office Workers—An Exploratory Analysis

**DOI:** 10.3390/ijerph17249158

**Published:** 2020-12-08

**Authors:** Viktoria Wahlström, David Olsson, Fredrik Öhberg, Tommy Olsson, Lisbeth Slunga Järvholm

**Affiliations:** 1 Department of Public Health and Clinical Medicine, Umeå University, 901 87 Umeå, Sweden; david.olsson@umu.se (D.O.); tommy.g.olsson@umu.se (T.O.); lisbeth.slunga-jarvholm@umu.se (L.S.J.); 2 Department of Radiation Sciences, Umeå University, 901 87 Umeå, Sweden; fredrik.ohberg@umu.se

**Keywords:** technical measurements, occupational health, office design, sedentary behavior, variance, workplace

## Abstract

Studies using technical measurements of physical behavior show wide interindividual variations. This study aimed to explore underlying factors related to sitting, standing and walking among office workers. Cross-sectional data for background characteristics, work-related variables, and device-based measures for sitting, standing and walking were collected among office workers in either a cell office or a flex office with activity-based work. Data were analyzed by Factor Analysis of Mixed Data (FAMD) and multiple robust linear regression. The FAMD resulted in the combination of underlying factors describing six character types. The (1) harmonic and healthy, (2) disabled with poor health, (3) manager that spend a lot of time in meetings and has very high workload, (4) engaged with high workload, (5) employee with creative and computer intense work, with high workload and, (6) employee with high BMI with creative and collaborative work. Regression analysis showed that the character type that was “engaged with high workload” sat more and stood less, while the character type with ”high BMI and with creative and collaborative work” sat less. The results suggest that physical behavior among office workers is influenced by a complex combination of factors, which should be taken into account in the evaluation of future studies of larger cohorts.

## 1. Introduction

Due to the technical developments in society, the percentage of sedentary occupations has increased during the last 50 years [1]. Notably, office workers could spend up to three-quarters of their worktime seated [2,3,4,5] and excessive sitting may increase the risk of obesity, heart disease and premature death [6,7,8]. There is therefore a need for intervention studies aiming to change physical behaviors towards less sitting and more physical activity, such as standing and walking in this workgroup [9].

In office settings, many factors could influence patterns for physical behaviors. Qualitative studies have identified barriers and facilitators for decreasing sitting and increasing standing and walking in office settings. Social and cultural norms, leadership, work tasks, workload and musculoskeletal disorders have been described to impact physical behaviors at work [10,11,12,13]. Environmental factors like building and interior design have also been described as important for physical behaviors [13,14]. Thus, interventions to decrease sitting and increase standing and walking in offices are suggested to target environmental, organizational, group and individual levels [9,15,16]. Intervention studies in office settings have shown promising results regarding the possibility to reduce time in sitting and increase standing [2,15]. Sedentary behavior (SB) is defined as any waking behavior with an energy expenditure ≤1.5 metabolic equivalents, in a lying, reclining or sitting posture [17]. Studies using technical measurements of SB and physical activity have shown wide standard deviations indicating large interindividual variations [13,18], but the putative underlying factors for these variations are not yet fully explored [5,19,20].

An exploratory study by Hadgraft et al. [19] using technical measurements of sitting, standing and walking among office workers showed a variation in sitting time between worksites. The study also shows that employees with more than five years of organizational tenure spent more time in total and prolonged sitting (>30 min), compared to those employed for 3–5 years. Notably, subjects with a high body mass index (BMI) (obese) spent less time in total and prolonged sitting time compared to normal-weight individuals. Another study examined whether the social–cognitive constructs, like knowledge and self-efficacy barriers, mediated intervention effects of a multicomponent intervention and found that variation in sitting reduction was only to a small extent explained by perceived behavioral control and individual self-efficacy barriers [21]. Sugiyama et al. [22] found that employees at worksites with mainly phone-based work or with mixed work tasks sat more compared to employees at worksites with non-phone-based work.

It is proposed that the office design could influence physical behaviors among office workers [23]. In recent years, flex offices with activity-based work (ABW) have increased in popularity [24,25]. Flex offices provide different workspaces for the employees. Workstations in landscape areas are complemented with support areas: cell offices for secluded work, meeting rooms in different sizes, lounges and break spaces. Employees do not have personal workstations and depending on the current work task, they are expected to choose a suitable space for the work task at hand [26].

As described, several factors might putatively influence physical behaviors in office settings, however, only a few studies have explored factors for these behaviors in office settings by using technical measurements of physical behaviors combined with a broad range of possible explaining factors [20]. These variations should be further explored in order to improve the efficacy, and evaluation of future workplace interventions focusing on decreasing sitting in office settings. The aim of the current study was to explore the underlying factors related to sitting, standing and walking behaviors among office workers in two different office types.

## 2. Materials and Methods

### 2.1. Design, Recruitment and Participants

We previously presented results from a multicomponent intervention study among office workers who relocated to different office types; one group to a cell office and the other to a flex office with ABW [27]. In short, results showed that employees sat for about 50% and stood for about 40% of the time at work in both groups already at baseline, which is lower levels of sitting and higher levels of standing compared to other studies. No changes in sitting or standing time were observed after the intervention. However, the time spent walking increased in the group moving to the flex office. Similar to other studies, our data also showed wide interindividual variations [19,27].

Participants were recruited among office workers in a public administrative workplace in northern Sweden. The Active Office Design (AOD) study had a longitudinal design with follow-up of employees in two groups, where one group moved to a new flex office, and the other to a new cell office [27]. Employees in departments for economy, human resources, urban planning, education, and politicians, relocated to the flex office with ABW, while the welfare office including social workers moved to a building with a cell office design. We collected repeated technical measurements for sitting, standing and walking, questionnaire data on lifestyle and health, detailed information on work tasks, and work-related psychosocial factors. Detailed information about work tasks was collected at 18 months after relocation. These assessments provided novel opportunities to perform exploratory analyses of underlying factors for physical behaviors among office workers. To be included for technical measurements of physical behaviors employees should be between 18–63 years, work 75% or more, and spend more than 60% of work time in the office. The participants were recruited following a computer-generated list, based on all employees included in the office relocation. The randomly selected employees were contacted via an e-mail invitation followed by a phone interview. Originally 86 participants were recruited for the study. At follow-up at 18 months after relocation, there were 59 participants left in the study. In the current study, we performed a secondary analysis, using cross-sectional data collected at 18 months after relocation. We finally included 53 participants with complete data. The reasons for drop-out were related to parental leave, sick leave, retirement or relocation to another office. More details on the study design, recruitment and reasons for drop-out are described elsewhere [27]. Employees were informed that participation was voluntary and that they could withdraw participation at any time according to the Declaration of Helsinki. All participants signed informed consent prior to participation. Ethical approval was granted by the Regional Ethical Review Committee in Umeå, SE (No: 2014/226-31).

### 2.2. Setting

In both offices, all workstations had sit-stand tables and adjustable chairs. The flex office was equipped with sit-stand tables in some meeting rooms, and both sitting and standing tables in the break spaces. Waste-paper bins and printer rooms were centrally placed on each floor, and 16 treadmills were placed in the flex office. In the cell office, each floor had corridors with personal cell offices along the outer walls. In the middle, there were shared surfaces like meeting rooms, break spaces and archives.

Both before and during the study, the studied organization, including both the cell office and the flex office group had an ongoing systematic health-promoting program, including, i.e., subsidized gym fees, annual step-count competitions, and bicycles were available for transport to meetings outside the office. The organization had a policy that employees could exercise for one hour a week during working hours (common in Sweden). For both office groups, a multicomponent PA promoting program, including environmental, organizational-, group- and individual-level components was implemented prior to the follow-up at 18 months. The intervention program has been described in detail elsewhere [27].

### 2.3. Measures

#### 2.3.1. Background Characteristics and Personal Health

In the questionnaire, participants reported age, gender, office type, and employment rate. Self-rated general health was assessed by a question from the SF-36 Short-form Health Survey which has shown strong reliability and validity [28]. Musculoskeletal symptoms from different body regions were reported by five items on a five-graded scale from never to always [29]. Mental disorders like stress and concentration difficulties were reported by two items on a five-graded scale from never to always [29]. Quality of sleep was reported on a five-graded scale between “very good” to “very bad” using the question “How do you perceive your sleep overall?” from the Karolinska Sleep Questionnaire [30]. Exercise habits were reported using the question “How often have you worked out or exercised in training clothes during the last 3 months, to improve your fitness and/or your wellbeing?” and answers were provided on a five-graded scale from never to >3 times per week [31]. Measurements of height and weight were performed at the workplace 18 months after relocation. Participants wore underwear, and standardized methods were used [32]. Body mass index (BMI) was calculated by using the body height, measured to the nearest 0.1 cm with a wall-mounted stadiometer (Hyssna 4146, Hyssna Measuring Equipment AB, Hyssna, Sweden), and body weight measured the nearest 0.1 kg using a calibrated electronic digital scale (Tanita BWB-800 MA; Umedico AB, Rosersberg, Sweden).

#### 2.3.2. Work-Related Variables

Measures for both work tasks and psychosocial work situations were assessed in the questionnaires.

Participants reported the total number of work hours per week and the number of hours per week spent in both small (2–3 persons) and large meetings (>3 persons). Based on this, meeting participation was calculated to a relative value per week. The number of meetings outside the office within walking or cycling distance was reported on a six graded scale from “never” to “daily”. Hours of computer-based work per day were reported on a four graded scale (from 0–1 h to 6–8 h per day). Work requirements, like the need to discuss with colleagues, to be creative or fully concentrated, were assessed by eight items on a five-graded scale from “never” to “always”. This instrument has not been validated but was developed by the researcher Helena Jahncke to further understand the content of work tasks related to office design. The instrument is based on results from laboratory studies, identifying work tasks and processes affected by noise in open landscape offices [33,34].

To measure experiences of work and the workplace we used the Work Experience Measurement Scale (WEMS) [35], which includes 32 statements grouped into six dimensions; (1) Supportive working conditions, (2) Internal work experience, (3) Autonomy, (4) Time pressure, (5) Management and (6) Reorganization. Each statement was assessed on a six-graded scale from “Do not agree at all” to “Totally agree”. WEMS has been designed to measure specific work-related aspects and salutogenic work factors. It is developed from theories related to a sense of coherence, demand/control/support, flow, and effort/reward.

For assessments of physical, mental and social well-being we used the Salutogenic Health Indicator Scale (SHIS) [36]. Twelve statements were assessed on a six-graded scale. An example of a statement could be, “During the last four weeks I have: (1) felt full of ideas and been creative, to (6) had a lack of ideas and creativity. Results from the 12 statements were combined into two dimensions; “Intrapersonal traits” (IPK) capturing perceived personal health state and the “interactive function” (IAF) measuring coping and interaction on requirements related to the working environment. Both for SHIS and WEMS results from all questions were weighted as a total index. The result was then standardized to a scale from 0 to 100. A high value indicates a high degree of positive experiences from work (WEMS) and perceived health (SHIS) [36]. Cognitive stress was measured using four questions from the second version of the Copenhagen Psychosocial Questionnaire (COPSOQ II), where problems with the ability to concentrate, make decisions, remember things or solve problems, was reported on a five-graded scale from never to always. In the analysis, the mean value of these four items was used [37]. The workload was assessed by the question “What are your thoughts on your workload?” and was reported on a six-graded scale from “too low” to “so heavy that I am bordering on exhaustion”.

#### 2.3.3. Technical Measurements of Physical Behavior—Sitting, Standing and Walking

Technical measurements of physical behaviors were performed over seven consecutive days. Participants wore ActivPAL3 or the ActivPAL3 micro (PAL Technologies Limited, Glasgow, Scotland; default settings) on the right thigh for 24 h per day for one week. ActivPAL provides valid, reliable, and sensitive measurements for time spent sitting, standing and walking [38,39]. Data were processed using a custom Excel macro (HSC PAL analysis software v2.19s, developed by researcher Philippa Dall and Malcolm Granat, Glasgow, UK). By using a logbook during the week of measurement, participants noted what time they got up, went to bed, started and finished work, whether it was a work or non-workday, periods of non-wear time or adverse events related to the measurements. The logbook was used to distinguish between work time and total awake time on workdays. In this study, we used data from work time only. To be eligible for analysis, the measurement period had to include at least 3 workdays and ≥4 h of work time was required [40]. The activity outcomes were standardized to an 8 h workday. Time spent in sitting >30 min bouts was defined as the time in prolonged sitting.

### 2.4. Statistical Analysis and Process for Result Interpretation

Before any analyses, all scales in the questionnaire were adapted so that high numbers corresponded to a high value. All continuous variables were standardized by transforming to a mean value of zero and a standard deviation of one.

**Figure 1:**
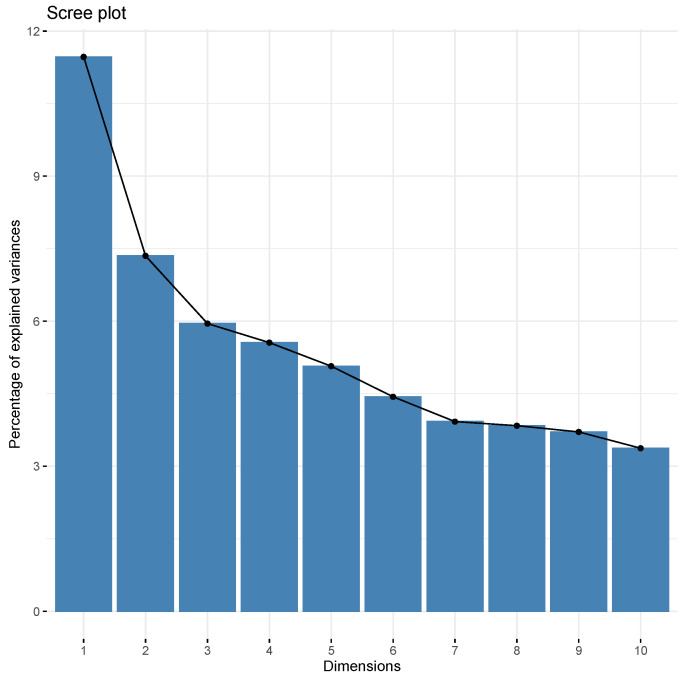
The Scree plot of the factors. The first six factors were further analyzed.

We combined Factor Analysis of Mixed Data (FAMD) with multiple linear regression. FAMD is a generalized principal component method that allows for both categorical and continuous data. Just like principal component analysis, FAMD aims to reduce the number of factors in the dataset while retaining as much of the variation as possible from the original dataset [41], and is a useful multivariate data analysis method to identify patterns within variables. The factors will contain combinations of variables, and the factors emerging will be uncorrelated. This means that we will not introduce bias in a regression model, or inflate the standard error estimates by including the factors as separate explanatory variables. Thus, allowing us to simultaneously assess any association between our explanatory variables, physical behaviors and office type without the loss of statistical power. In the first step, we performed a FAMD on the background characteristics, personal health and work-related variables to reduce the number of factors in the dataset. Each factor contained unique combinations of personal health (i.e., self-reported health, sleep, pain), factors related to work tasks (i.e., time spent in meetings, amount of computer work and work requirements), and the psychosocial work situation (data from WEMS, SHIS, COPSOQC). We used a scree plot to determine the number of factors that should be kept for the analyses, based on where the Scree plot leveled out (Figure 1). The combinations of variables in each factor were interpreted by two of the authors, first separately and then together. The interpretation was based on the explanatory variables that fell out significant in each factor. The higher degree of explanation of variation a variable had, the more impact on the interpretation. The combination of exploratory variables emerged from the FAMD and the factors could be described as the base for “animated” employees. The explaining variables were lastly verbally condensed to describe character types. 

In the second step, we performed multiple robust linear regression analyses to explore associations between the character types (factors), the ActivPAL data on sitting, standing and walking and office type. In the linear regression analyses, we decided to report all estimated associations between character types (factors) and data for sitting, standing and walking per interquartile range increase (IQR), where IQR is defined as the difference between the 75th and 25th percentile. We used an interquartile range instead of a unit increase, since a unit increase in any given character type does not have a specific meaning, meanwhile, an interquartile range increase at least gives the magnitude of the effect for a given character type. The statistical significance was determined by 95% confidence intervals. The FAMD analysis was performed using the “FactoMineR” and “factoextra” packages and the multiple robust linear regression models were fitted using the “MASS” package in R computing software, version 3.5.2 (R Foundation for Statistical Computing, Vienna, Austria). For descriptive statistics of sample characteristics, we used SPSS software v.24 (IBM Corp, Armonk, NY, USA).

## 3. Results

### 3.1. Participant Characteristics

The characteristics of participants are presented in Table 1. Approximately 60% of the participants exercised ≥2 times per week and 40% exercised once a week or less. On average participants spent 53% of their worktime sitting, 38% standing and 9% walking.

### 3.2. Factor Analysis of Mixed Data

For each factor, the FAMD resulted in a complex combination of factors. These factors together explained 31% of the total variance in the data. The combination of significant variables within the factors resulted in a description of six character types that were named (1) harmonic and healthy, (2) disabled with poor health, (3) manager that spend a lot of time in meetings and has very high workload, (4) engaged with high workload, (5) creative and computer intense work, with high workload, and, (6) had high BMI and creative and collaborative work. The list of the significant variables and the characters types is presented in Table 2.

### 3.3. Regression Analysis

In the second step, we explored how the different character types and office types related to activity outcomes for sitting, standing, walking (Table 3). The character type “engaged with high workload”, stood less and spent more time in both total and prolonged sitting, when in the fourth quartile compared to the first quartile. The character type with “a high BMI with creative and collaborative work” sat 34 min less and stood 30 min more, where the sitting time was significant (Table 3). The “harmonic and healthy” seemed to sit more and stand less, but this was not significant. Office type did not affect the level of sitting, standing or walking at work. The variables included in the regression models explained 20.8% of the variation in standing time and 12.8% of walking time variation. For sitting and prolonged sitting, 22.3% and 19.3% of the variation was explained.

## 4. Discussion

The aim of this study was to explore the underlying factors related to sitting, standing and walking among office workers in two office types by using device-based data on physical behaviors, and a broad range of possible explaining factors. Our results revealed that a complex combination of factors related to personal health, work tasks and psychosocial work situation influenced patterns for sitting and standing at work. These results suggest that office workers are a heterogeneous group in these respects, even if they work within the same organization.

We found that the “engaged employee with high workload” spent more time in total and prolonged sitting. Exploring data from a previous cluster-randomized trial, Hadgraft et al. [19] found large differences in sitting time between worksites. When analyzing data from the same trial, Sugimaya et al. [22] found that the level of task variation differed between teams, and those with a high degree of phone-based work spent more time sitting than teams with other tasks. Our results suggest that both work engagement, workload and type of work tasks seem to influence total sitting time and the way sitting time is accumulated at work.

The character types that were highly “harmonic and healthy” had a tendency to sit more and stand less, although not statistically significant. To our knowledge, there are no previous studies with similar exploratory results, although a qualitative study by Flint et al. [42] reported that regular exercisers or those with a young family, which could be presumed to have good health, were less motivated to reduce their sedentary time at work. In line with these results, Nooijen et al. [43] found that a higher proportion of young workers reported that they thought standing was uncomfortable and tiring, and were less motivated to stand at work, and a higher proportion of men reported regular exercise as a barrier to standing at work. The results from Flint et al. and Nooijen et al. [42,43] might indicate that individuals with good health might be less worried about the health risks of excessive sitting, and therefore tend to sit more at work, which is in line with our results.

In our study, the character type with “high BMI and creative and collaborative work” sat less. Hadgraft et al. [19] found that employees with high BMI sat less compared to those with a normal BMI. The “manager that spend a lot of time in meetings and has very high workload” did not sit or walk more than other character types, even though they spent more time in meetings and more time outside the office. This might be due to managers’ high autonomy in their performance of work tasks.

In a study investigating health beliefs and attitudes among office workers, Sudholz et al. [44] found that employees and employers associated occupational sitting more with musculoskeletal disorders and performance-related concerns rather than long-term health effects. Intervention studies where participants have decreased their sitting and increased their standing time have shown improvements in musculoskeletal disorders [45,46,47]. In our study “the disabled employee with poor health” did not seem to sit more than other character types, and the average standing time was high during the whole study period [27]. This may indicate that interventions to reduce sitting and increase standing, successfully could reach out to individuals that would gain mostly with positive health effects. Individuals with musculoskeletal disorders might also choose to vary between sitting and standing to a greater extent to increase variation as a way to cope with their symptoms.

As the work tasks and the psychosocial work situation of office workers can vary a lot, the local context is of importance when developing interventions. Previous studies recommend multicomponent interventions, targeting both environmental-, organizational-, group-, and individual levels [23,48]. To be able to address specific barriers and enhance possibilities to decrease sitting and increase standing and walking in an organization, a recent study emphasizes the need for a participatory approach when designing interventions [16]. The participatory approach creates an opportunity to consider local contexts, like work tasks, the health-promoting culture and physical environment. The results of this study indicate that office workers have different physical behaviors depending on a complex combination of both health- and work-related factors. We recently published a study where the implementation process of the PA promoting program was evaluated using mixed methods [49]. The process evaluation was performed in the flex office group, representing 53% of the study population in the current study. The qualitative results showed that physical behaviors were contextual, influenced by many different factors and could vary substantially between individuals [49]. The results for the secondary analysis in the current study confirm the complex combination of underlying factors for physical behaviors among office workers. Based on our new results here presented, we would suggest that interventions might even need to be tailored on a group level within organizations, i.e., different departments, to be more realistic and specific, which might lead to improved effects.

A limitation of this study is the cross-sectional design which restricts the possibility to assess how different variables change over time. The relatively small sample size, with only a few men and managers, also lowers the generalizability of the results. The working hours, culture, norms and physical environment in offices could also differ between countries. In our study, the organization had a strong tradition to promote a healthy lifestyle and physical activity, and all workers already had sit-stand tables, which decrease the possibility to generalize our results to other office populations. Further, the data analyzed in this study were collected shortly after the implementation of a multicomponent PA promoting program, aiming to decrease sitting and increase standing and walking. Altogether, this suggests that the results should only with caution be generalized to other office populations. We previously presented results from the AOD Study that showed significant interaction effects in walking time and number of steps over time between employees working in the cell versus flex office [27]. In the current study, the office type did not seem to be related to behaviors for sitting, standing or walking, which might be caused by loss of power due to the cross-sectional design.

Strengths of this study include the technical measures for sitting, standing and walking, combined with a broad range of questionnaire data on background characteristics, personal health and work-related variables. This enables an increased understanding of the complexity of factors of importance for physical behaviors in office settings. The advantage of using a generalized principal component method before analyzing the outcomes is that the number of explanatory variables is reduced. This makes it feasible to explore the association between a wide range of putative explaining variables and the data on physical behaviors. However, this methodology complicates the interpretation of the regression analysis, as it is not obvious exactly what the resulting factors represent.

Even though the interpretation of our results should be carried out with caution, our results indicate that physical behaviors among office workers are associated with complex combinations of explanatory variables. This complexity suggests that many aspects are to be considered both when planning interventions and evaluating effects from this type of organizational interventions. In addition to detailed descriptions of the worksite culture and physical environment, also workload, health variables such as BMI, musculoskeletal disorders, amount of computer work, and specific work requirements could be possible mediators in an intervention. Therefore, data collection with a broad perspective on work factors is important in future studies.

## 5. Conclusions

We found that complex combinations of factors influence physical behaviors among office workers. The interindividual variation of time spent sitting, standing and walking were only partly explained by our multifactorial approach. More studies using technical measures for physical behaviors, combined with assessments of a broad range of possible underlying factors should be taken into account in the evaluation of future studies of larger cohorts.

## Figures and Tables

**Table 1:** Characteristics of participants, *n* = 53.

	Mean (SD)	*n* (%)
Background and lifestyle variables		
Female		44 (83)
Age	52.3 (9.4)	
BMI	26.2 (3.7)	
Physical exercise		
No exercise		6 (11)
Occasionally—not regular		9 (17)
1 time per week		6 (11)
2–3 times per week		23 (44)
>3 times per week		9 (17)
Health variables		
Self-rated general health		
Very good or excellent		29 (55)
Good		19 (36)
Fair or bad		5 (9)
Sleep disorders ^a^		
Never or seldom		28 (54)
Sometimes		16 (31)
Often or always		8 (15)
Discomfort from neck and shoulders ^b^		
Never or seldom		18 (35)
Sometimes		16 (31)
Often or always		17 (33)
Work-related variables		
Managerial position		8 (15)
Computer work per day		
0–2 h		0(0)
2–4 h		5 (9)
4–6 h		20 (37)
6–8 h		28 (53)
Workload ^a^		
Too low		0 (0)
Adequate, never bothersome		2 (4)
Sometimes bothersome		27 (52)
Somewhat bothersome		9 (17)
Often bothersome		10 (19)
So heavy that I am bordering on exhaustion		4 (8)
Office type		
Flex office		28 (53)
Cell office		25 (47)
Activity outcomes per 8-h workday		
Total sitting time per day, %	53.1 (17.0)	
Time spent in prolonged sitting per day, %	22.7 (12.1)	
Total standing time per day, %	37.5 (16.7)	
Total walking time per day, %	9.4 (3.2)	

^a^ = data missing from 1 participant, ^b^ = data missing from 2 participants.

**Table 2:** Presentation of the character types, based on variables of influence from the factor analysis of mixed data. Covariates and factors for each character type are presented in descending order, based on r2.

Character Type	Condensed Characteristics	Significant Variables of Influence, Presented in Descending Order	Level	Correlation (r), Scale Data	Estimated r^2^, Ordinal Data
“The harmonic and healthy employee”	The harmonic employee with good health and high wellbeing. No problems with stress, sleep, pain or concentration.	Interpersonal traits ^a^		0.78	
		Interactive function ^a^		0.77	
		Cognitive stress		−0.72	
		Symptoms of stress	Seldom or never		0.47
		Concentration disorders	Seldom or never		0.44
		Sleep quality	Very good		0.42
		Concentration demands	Medium		0.40
		Exhaustion disorder	No		0.37
		Discomfort in neck and shoulders	Seldom		0.35
		Need to be fully concentrated at work	Seldom		0.35
		Perceived support at work		0.57	
		Self-reported health	Very good		0.32
“The disabled employee with poor health”	The disabled employee with poor health, musculoskeletal disorders, high BMI, concentration difficulties and high stress levels. Work requires high creativity, a need to learn new things and to discuss with colleagues.	Discomfort in hip, knee or feet	Always		0.60
		Discomfort in back	Always		0.58
		Discomfort in neck and shoulders	Always		0.39
		BMI		0.62	
		Weight		0.57	
		Concentration disorders	Often or always		0.26
		Need to learn new things at work	Sometimes		0.24
		Self-reported health	Fair or bad		0.23
		Need to be creative at work	Often		0.23
		Symptoms of stress	Always		0.22
		Need to remember things at work	Sometimes		0.21
		Need to discuss with colleagues	Sometimes		0.17
“Manager that spend a lot of time in meetings and has very high workload”	Managers working partly outside the office. Spends time in large and small meetings. Very high workload, but no sleep disorder. Work requires medium concentration demands, seldom works on the phone or handle secrecy.	Meetings outside the office	1–2 times/week		0.34
		Confidential work tasks	Seldom or never		0.31
		Time in large meetings		0.55	
		Time outside the office		0.54	
		Concentration demands	Medium		0.25
		Managerial position	Yes		0.23
		Workload	So high that I am boardering exhaustion		0.22
		Time in small meetings		0.46	
		Time in phone calls		−0.45	
		Sleeping disorders	Seldom or never		0.20
“The engaged employee with high workload”	The employee that perceives work tasks and support at work positive. High workload sometimes pain in neck and shoulders and concentration disorders, but not exhausted. Works outside the office, and by the computer for 4–6 h per day.	Perceived change management support ^b^		0.61	
		Internal work experience ^b^		0.57	
		Exhaustion disorder	No		0.28
		Workload	Somewhat bothersome		0.27
		Discomfort in neck and shoulders	Sometimes		0.21
		Concentration disorders	Sometimes		0.17
		Time outside the office		0.40	
		Computer-based work	4–6 h		0.17
		High appreciation of management ^b^		0.33	
“The employee with creative and computer intense work and high workload”	The employee with high workload and perceived high control. Works mainly computer bound inside the office, often in small meetings. Sleeps quite well. Work requires high creativity.	Sleep quality	Quite good		0.34
		Need to learn new things at work	Often		0.28
		Computer-based work	6–8 h		0.27
		Workload	Very high		0.24
		High autonomy at work ^b^		0.36	
		Time outside the office		−0.44	
		Sleeping disorders	Seldom or never		0.34
		Time in small meetings		0.39	
		Need to be creative at work	Always		0.13
”The employee with high BMI with creative and collaborative work”	The employee with a high but balanced workload. Exercise once a week, has a high BMI and seldom perceives discomfort in neck and shoulders. No need to learn new things at work, need to be creative, has high concentration demands and often works in small meetings.	Need to learn new things at work	Seldom		0.3
		Exercise habits	Once per week		0.27
		Need to be creative at work	Always		0.27
		Medium time pressure ^b^		0.5	
		Workload	Sometimes bothersome		0.23
		Discomfort in neck and shoulders	Seldom		0.21
		BMI		0.4	
		Need to be fully concentrated at work	Often		0.16
		Time in small meetings		0.31	

^a^ Salutogenic Health Indicator Scale, high value indicates good health, ^b^ Work Experience Measurement Scale, high value indicates a positive experience of working conditions.

**Table 3:** Results from robust regression analysis, showing the impact of character types and office type on activity outcomes. The effects of character types are reported per interquartile range (IQR) increase. IQR is defined as the difference between the 75th and 25th percentile of the factor scale. Statistically significant outcomes are marked in bold.

Activity Outcome and Character Name	Minutes/Day	95% CI
Total sitting time		
Flex Office (reference cell office)	−17	(−87, 52)
“The harmonic and healthy employee”	39	(−5, 83)
“The disabled employee with poor health”	−17	(−34, 1)
“Manager that spend a lot of time in meetings and has very high workload”	−9	(−37, 19)
“The engaged employee with high workload”	**32**	**(4, 60)**
“The employee with creative and computer intense work and high workload”	16	(−14, 45)
”The employee with high BMI with creative and collaborative work”	**−34**	**(−68, −1)**
Prolonged sitting time		
Flex Office (reference cell office)	−28	(−78, 22)
“The harmonic and healthy employee”	14	(−18, 45)
“The disabled employee with poor health”	−10	(−22, 2)
“Manager that spend a lot of time in meetings and has very high workload”	18	(−2, 38)
“The engaged employee with high workload”	**28**	**(8, 48)**
“The employee with creative and computer intense work and high workload”	6	(−15, 26)
”The employee with high BMI with creative and collaborative work”	−22	(−46, 2)
Standing time		
Flex Office (reference cell office)	16	(−57, 89)
“The harmonic and healthy employee”	−45	(−90, 1)
“The disabled employee with poor health”	15	(−3, 33)
“Manager that spend a lot of time in meetings and has very high workload”	6	(−23, 36)
“The engaged employee with high workload”	**−29**	**(−58, 0)**
“The employee with creative and computer intense work and high workload”	−12	(−42, 18)
”The employee with high BMI with creative and collaborative work”	30	(−5, 65)
Walking time		
Flex Office (reference cell office)	0	(−10, 11)
“The harmonic and healthy employee”	6	(−1, 12)
“The disabled employee with poor health”	0	(−2, 3)
“Manager that spend a lot of time in meetings and has very high workload”	2	(−2, 7)
“The engaged employee with high workload”	−3	(−7, 2)
“The employee with creative and computer intense work and high workload”	−3	(−8, 1)
”The employee with high BMI with creative and collaborative work”	4	(−1, 9)
